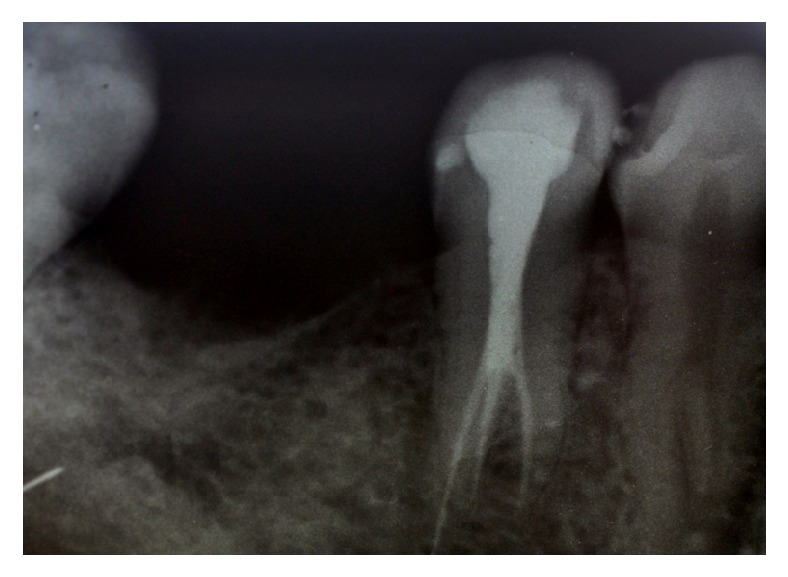# Endodontic Treatment of a Mandibular Second Premolar with Three Roots and Three Canals

**DOI:** 10.1155/2014/973410

**Published:** 2014-11-05

**Authors:** Bonny Paul, Kavita Dube

**Affiliations:** Department of Conservative Dentistry & Endodontics, Hitkarini Dental College & Hospital, Jabalpur 482005, India

## Abstract

Complex root canal system with atypical variations is a common finding among mandibular premolars. Endodontic treatment in these teeth may not be successful due to the failure to recognise and treat multiple canals. This paper presents endodontic treatment of a mandibular second premolar with three roots and three canals.

## 1. Introduction

A thorough knowledge of the basic root canal anatomy and its possible variations is essential for achieving successful nonsurgical endodontic treatment. Investigators have reported multiple foramina, fins, deltas, loops, furcations, and accessory canals in most teeth [1]. The main reasons of endodontic failures are apical percolation and the presence of microorganisms caused by incomplete cleaning, insufficient canal obturation, and presence of untreated canals [2].

Anatomically lower second premolars are described as teeth with single root and single root canal [3]. However they could be the most challenging to treat due to the failure to identify the complex variations in their root canal morphology. The ovoid shaped root in cross section normally has developmental grooves or depressions on the mesial and distal surfaces [4].

Root canal anatomy of mandibular second premolar can be highly complex. The purpose of this paper was to describe endodontic treatment of mandibular second premolar with three separate canals and three separate roots.

## 2. Case Report

**Figure 1:**
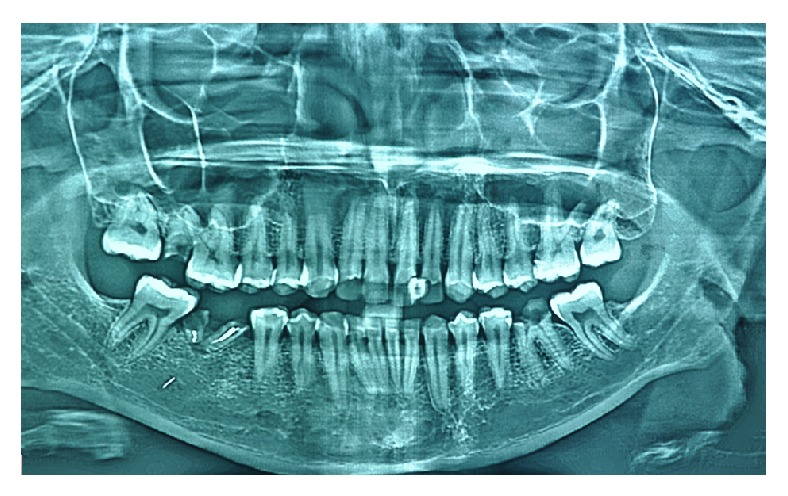


**Figure 2:**
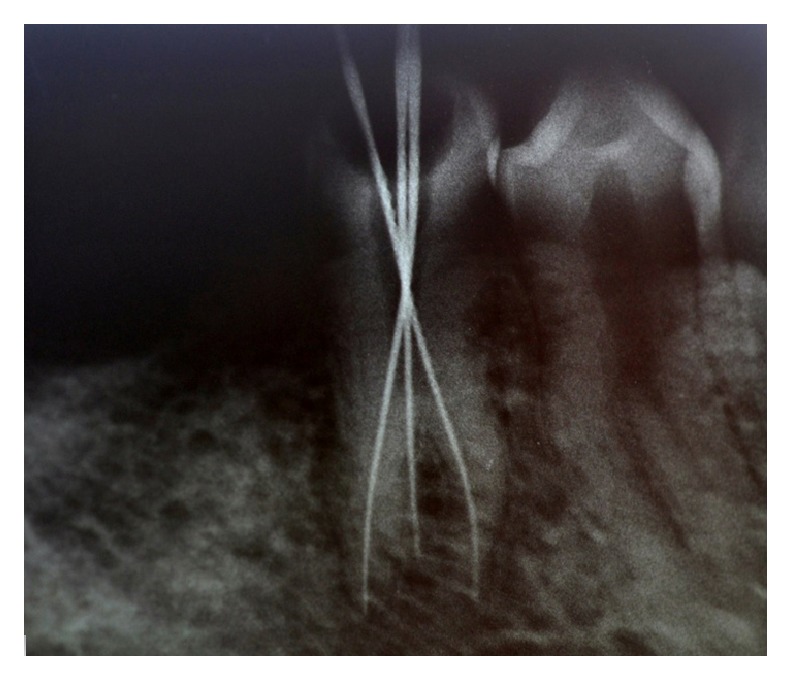


A 30-year-old male patient, with a noncontributory medical history, reported to the Department of Conservative Dentistry and Endodontics, Hitkarini Dental College and Hospital, Jabalpur, with pain in his lower right second premolar (45). The pain was spontaneous, increased on lying down, and present for the past 2 days. Clinical and radiographic (Figure 1) examination revealed a deep carious lesion in the same tooth and also presence of root stump of first molar. Vitality testing with dry ice (R C Ice, Prime Dental) caused severe lingering pain. A diagnosis of symptomatic irreversible pulpitis was made and it was decided to carry out endodontic treatment in second premolar and extraction of first molar root piece. Local anaesthesia was achieved by administration of inferior alveolar nerve block with 2% lidocaine; the premolar was isolated under rubber dam (Hygenic-Coltene Whaledent). Following excavation of caries a conventional access cavity was prepared with Endo Access bur FG1 (Dentsply Maillefer, Switzerland). Clinical examination with a DG 16 explorer (Hu-Friedy, USA) revealed three orifices. The access cavity was modified slightly to expose the three orifices. Working length radiographs (Figure 2) revealed three separate canals with three separate roots. The canals were instrumented with Flexo files (Dentsply Maillefer, Swiss made), using EDTA (Dentsply Maillefer, USA) as lubricant. An apical preparation till 30 no (2%) was carried out. The canals were irrigated using normal saline and 3% sodium hypochlorite (Vishal Dental products, India). After confirming the master cone (Dentsply, India) by radiographs the canals were dried using paper points (Dentsply, India) and obturated by lateral condensation technique using AH 26 (DeTrey/Dentsply, Germany) as sealer. A temporary dressing (Cavit G, 3M ESPE, Germany) was given and a radiograph was taken to confirm the obturation (Figure 3). The patient was recalled after a week and was found to be asymptomatic.

## 3. Discussion

Slowey felt that mandibular premolars were the most difficult teeth to treat endodontically because of their aberrant anatomy [5]. A complete awareness of their statistical data is important for the clinician to achieve a higher degree of success in endodontic treatment.

Studies have reported that the incidence of two or more canals in mandibular second premolar may vary between 1.2% and 34% [6–8]. Sert and Bayirli have reported an incidence of 0.4% of mandibular second premolars with three root canals [9]. Vertucci assessed the root canal morphology in 100 Turkish male and 100 Turkish female patients [10]. Men (43%) exhibited two or more canals much more frequently than the female patients (15%) in the study. Vertucci reported an incidence of 2.5% of two separate and distinct root canals in mandibular second premolars; however he has not reported any case of mandibular second premolar with three root canals [11].

Mandibular second premolars mostly have a single root. The incidence of 2 or more roots is low, approximately 0.4%, whereas in mandibular first premolar it is 2.1%. Majority of the mandibular premolars have a single canal, but approximately 9% have 2 or more canals. A single apical foramen might be found in mandibular teeth in more than 9 out of ten cases, but 2 or more foramina may occur approximately 8.2% of the time. The incidence of more than 1 root, more than 1 canal, and more than 1 foramen is less frequent in the mandibular second premolar than in the mandibular first premolar [12].

Albuquerque et al. reviewed the management of variable anatomy in mandibular premolars. They emphasized the importance of understanding of normal anatomy and common variations, careful interpretation of angled radiographs, use of three-dimensional imaging, proper access cavity preparation, and a detailed exploration of the interior of the tooth, ideally under magnification [13].

Thirty-six anatomic studies were analyzed by Kottoor et al. [14], which included 12,752 first premolars and nineteen studies assessing 6646 second premolars. A significant variation in the number of roots, root canals, and apical foramina was observed between Caucasian, Indian, Mongoloid, and Middle Eastern ethnicities. The most common anatomic variation was C-shaped canals in mandibular first premolars with highest incidence in Mongoloid populations (up to 24%).

The variability in root canal morphology is a usual phenomenon. Radiographs taken at different horizontal angulations facilitate searching for additional roots and canals. If a radiolucent line is present mesial or distal to the main canal, an additional canal should be suspected. Magnification and fibre optic illumination are helpful in increasing the optical field. Tactile examination of the walls of the major canal with a small precurved file tip is mandatory, even in cases which appear to have only one canal radiographically.

## 4. Conclusion

Successful endodontic treatment requires a detailed knowledge of root canal anatomy. The presence of extra canals should be thought of in every tooth undergoing endodontic treatment. This would help in reducing endodontic failure due to incomplete obturation.

## Figures and Tables

**Figure 3:**